# Supplementary material for: Machine learning classification reveals robust morphometric biomarker of glial and neuronal arbors
Source: J Neurosci Res. 2022 Oct 5;101(1):112–29. doi: 10.1002/jnr.25131 (PMC9828050; doi:10.1002/jnr.25131)
Supplement: Supplementary file 1 — Transparent Science Questionnaire for Authors [file JNR-101-112-s001.docx]

Rigorous study design and transparent reporting of results are the cornerstones of science. By maximizing the information provided in a manuscript, factors that may contribute to irreproducibility will be mitigated. The *Journal of Neuroscience Research* promotes transparency in research by strongly encouraging authors to include all relevant information about their studies (see our [preprint](https://osf.io/5cvqh/) for details). To expedite reviewer monitoring of these factors, authors submitting original research articles must complete this questionnaire.

If the manuscript is accepted and all items within the checklist are present, we will include a declaration of transparency at the end of the manuscript. This declaration reads as follows:

***The authors, reviewers and editors affirm that in accordance to the policies set by the Journal of Neuroscience Research, this manuscript presents an accurate and transparent account of the study being reported and that all critical details describing the methods and results are present***.

To complete the checklist, fill in the right-hand column with the page and paragraph number (e.g., ‘Page 3, Paragraph 2’) corresponding to the checklist item. If a checklist item is not applicable to the study being reported or the authors are unable to provide that item, a reason must be supplied. Additional comments can be added at the end of the document. Upload the completed document as supplementary information for review.

| Experimental and Study Design | Can we differentiate neurons and glia based on their morphometric features?  This hypothesis is mentioned on page 3, line 60-63. |
| --- | --- |
| 1. Clearly state the primary and any secondary objective or hypothesis of the study |  |
| 1. For each experiment, the study design must include:    1. Number of experimental and control groups    2. Randomization and blinding procedures and/or steps to minimize subjective bias when allocating subjects to experimental groups    3. Precise details of all procedures, including housing and husbandry are carried out in the experiment    4. Is sex considered as a biological variable? See [Editorial](https://pericles.pericles-prod.literatumonline.com/doi/full/10.1002/jnr.23979) for details about proper reporting | We downloaded a total of 31,370 cells, 15,690 neurons and 15,680 glia, from NeuroMorpho.Org which hosts the digital reconstructions of the nervous system cells from peer reviewed publications. This information can be found on page 6, line 127-128 and page 7, line 170-171. |
|  | We used the data of neurons and glia from already published articles in our study. More details can be found on page 5, line 112-128 of the manuscript. |
|  | The data was downloaded from an online repository called NeuroMorpho.Org and supervised classification was applied to see the geometric differences between neurons and glia. This is described in detail on page 4, line 87-93 of the manuscript. |
|  | Yes, the dataset we used included neurons and glia from male and female animals. The classification based on the sex and their results are mentioned in the article on page 16, line 385-399. |
|  |  |
| **Experimental Subjects** |  |
| 1. Specify the total number of subjects in each experiment, including the number of animals, sex and age in each group    1. Explain how the number of animals were arrived at and provide details of any sample size calculation, including power analysis    2. Indicate the number of independent replications of each experiment, when applicable | Since we used the already published data, some of the experiments did not mention these details and therefore we do not have an actual number of subjects, age, sex, etc. Details about the missing sex information can be found on page 16, line 387. |
|  | N/A |
|  | N/A |
| **Data Handling** |  |
| 1. Indicate data collection start and stop rules:    1. Define the criteria for data/subject inclusion and exclusion. If any outcome or condition measure used was not reported in the results section, authors must address this omission    2. Specify reasons for any discrepancy between the number of animals at the beginning and end of the study    3. Define and explain how outliers are handled and report if data are removed prior to analysis | We used all glia published in version 7.1 to 8.0 of NeuroMorpho.Org and balanced it with equal number of neurons with similar metadata as described on page 6, line 127-128. |
|  | N/A |
|  | No outliers were removed in this analysis. Two of the 21 morphometric features were removed called Soma Surface and Depth because some neurons and glia had null values for these features. The details about the removal of these morphometric features can be found on page 6, line 139. |
|  |  |
|  |  |
| **Statistical Analysis and Depiction of Continuous Data** |  |
| 1. Provide details of the statistical methods used for each analysis    1. State, define and justify the statistical analysis used and specify the unit of analysis for each dataset    2. Describe and report methods used to assess whether data met the assumptions of the statistical approach and any adjustments for multiple comparisons    3. Fully report statistics (including exact value of N, degrees of freedom, test value and exact P-value when >0.001) and we encourage the use of effect sizes and confidence intervals    4. Disaggregated data are presented for males and females    5. Data distribution is depicted with univariate scatterplots boxplots, violin plots, or kernel density plots when presenting **continuous data** (see Editorial [Publishing Transparent and Rigorous Scientific Research](https://osf.io/5cvqh/)) | We used dimensionality reduction, supervised machine learning methods (K-nearest neighbor, random forest, support vector machine), and PSwarm optimization techniques in this study. The details of these methods can be found on page 8-10, line 177-246. |
|  | K-fold cross validation was used for classification for the validation of results and is described on page 10, line 228-234 in the manuscript. |
|  | Confidence intervals for the Average Branch Euclidean Length (ABEL) is reported in the study and is shown in Figure 8B. All classification results are reported with accuracy, specificity, and sensitivity which can be found on page 13, line 317-323 and Figure 6. |
|  | Disaggregated data for males and females are presented in this classification. The details of this can be found in the manuscript on page 16, line 385-399. |
|  | Data distribution is depicted with violin plots which are shown in Figure 7. |
|  |  |
|  |  |
| **Discussion** |  |
| 1. Comment on study limitations including any potential source of bias, limitations to the animal model, imprecisions associated with the results, and the inability for any reason to study possible sex influences where they may exist. 2. Comment on possible translational implications and future research directions | One limitation of this study is when it comes to the classification of neurons and glia from cerebellum. Almost all granule neurons and Purkinje neurons were misclassified as glia, and all transitional oligodendrocytes were misclassified as neurons. The details about Cerebellum and ABEL limitations are described on page 17, line 410-416. |
|  | Future research directions include the classification of three main types of neurons and glia as a multiclass classification which is not possible now because of the imbalance of data among several classes. More details can be found on page 18, line 437. With the discovery of new morphological biomarker, it would be very interesting to see if ABEL can be used as a potential morphological biomarker in the identification of different cell types as explained in the manuscript on page 19-20, line 471-487. |
