# Supplementary material for: Machine learning classification reveals robust morphometric biomarker of glial and neuronal arbors
Source: J Neurosci Res. 2022 Oct 5;101(1):112–29. doi: 10.1002/jnr.25131 (PMC9828050; doi:10.1002/jnr.25131)
Supplement: Supplementary file 2 — Data S1 [file JNR-101-112-s002.docx]

**Supplementary Materials**

The following files are available at https://github.com/Masood-Akram/Classification_Neurons-Glia/tree/main/Supplementary_Material

Scale Correction Main Dataset: calculations of the correction factors for the archives of the main dataset reporting reconstruction coordinates in pixels rather than microns.

Scale Correction Additional Dataset: calculations of the correction factors for the archives of the additional dataset reporting reconstruction coordinates in pixels rather than microns.

Metadata Dimensions Main Analysis: detailed breakdown of the metadata for all archives of the main dataset.

Metadata Dimensions Additional Analysis: detailed breakdown of the metadata for all archives of the additional dataset.
 Male Female ABEL: detailed values of ABEL divided by cell type (glia and neuron) and the sex of the animal (male and female).

Sub-Type Classification: detailed values of ABEL divided by cell sub-type (microglia, astrocytes, oligodendrocytes, principal cells, interneurons, and sensory neurons), their distribution plots, and confusion matrix from 6-class SVM classification with sensitivity and specificity values.
